# Selected Metabolic Markers in Girls with Turner Syndrome: A Pilot Study

**DOI:** 10.1155/2018/9715790

**Published:** 2018-08-29

**Authors:** E. Błaszczyk, M. Lorek, T. Francuz, J. Gieburowska, A. Gawlik

**Affiliations:** ^1^Department of Pediatrics and Pediatric Endocrinology, School of Medicine in Katowice, Medical University of Silesia, Katowice, Poland; ^2^Students' Scientific Association at the Department of Pediatrics and Pediatric Endocrinology, School of Medicine in Katowice, Medical University of Silesia, Katowice, Poland; ^3^Department of Biochemistry, School of Medicine in Katowice, Medical University of Silesia, Katowice, Poland

## Abstract

**Background:**

Turner syndrome (TS) predisposes an individual to obesity and related metabolic disorders. As the TS population is at a higher risk of cardiovascular diseases and malformations, research into laboratory markers of metabolic complications has been ongoing. Special significance has recently been attributed to matrix metalloproteinases (MMPs), their inhibitors (TIMPs), and neurotrophic factors, such as BDNF and GDNF.

**Objective:**

To establish whether cardiometabolic risk in patients with TS is reflected in the concentrations of metalloproteinases and neurotrophic factors.

**Method:**

The concentrations of circulating MMP-1, MMP-2, MMP-9, TIMP-1, BDNF, GDNF, and VEGF were measured in 17 patients with TS. The control group was composed of 11 girls with nonpathologic short stature and normal karyotype.

**Results:**

There were no differences in chronological or bone age. No significant differences were observed in mean weight, although the *Z*-score BMI was higher in the study group. The mean baseline values of MMP-1 and BDNF were significantly lower in the control group than in the study group (*p* < 0.001, *p* = 0.001). Regression analysis revealed a positive correlation between MMP-1 concentrations and *Z*-score BMI (*r* = 0.36, *p* = 0.047) and between BDNF and *Z*-score BMI (*r* = 0.48, *p* = 0.013).

**Conclusion:**

Our pilot study showed that MMP-1 may be a potential indicator of a higher risk of cardiometabolic complications in girls with TS. The elevated concentrations of BDNF in normal-weight girls with TS need to be studied further, taking into consideration the influence of estrogen-androgen imbalance.

## 1. Introduction

Cardiovascular diseases are more likely associated with certain genetic disorders, including Turner syndrome (TS) [1]. Not only is obesity a major risk factor for many interrelated metabolic disorders, but it also leads to the development of cardiovascular diseases and, ultimately, to increased mortality [2]. Numerous studies indicate that metabolic syndrome components, including obesity and excessive waist circumference, occur more frequently in TS and that the phenomenon starts in childhood [3]. It remains unclear whether cardiometabolic and vascular risks in TS are the consequence of unidentified intrinsic factors or, conversely, the result of modifiable risk factors, such as obesity. New markers that could explain the pathogenesis of metabolic complications are under investigation.

*Brain-derived neurotrophic factor* (BDNF) is a member of the nerve growth factor family. It plays an important role in the modification of synaptic efficacy [4] and enhances neuronal survival [5]. Studies on rats have shown that BDNF is involved in weight regulation and energy expenditure by reducing appetite in rodents [6]. The concentration of this growth factor correlates negatively with body weight [7] and age in humans [8]. Moreover, patients who meet the metabolic syndrome criteria [9] and patients with acute coronary syndrome [10] have significantly lower plasma BDNF levels. These findings gave rise to the metabotropic deficit hypothesis [11], according to which the absence of neurotrophins can lead to the development of metabolic diseases [12].

*Glial cell line-derived neurotrophic factor* (GDNF) is a member of the neurotrophin family, and it is responsible for neuronal survival by protecting neurons from damage [13]. Due to its neuroprotective role, GDNF has been tested in the treatment of Parkinson's disease. In a study on monkeys with parkinsonism, one of the main side effects of intracerebroventricular injections of recombinant human methionyl-GDNF was weight loss [14]. A similar effect was obtained in humans [15]. This seems to support the metabotropic hypothesis.

The *vascular endothelial growth factor* (VEGF) family includes VEGF-A, -B, -C, -D, -E, and -F and placental growth factor (PGF). VEGFs are involved in the formation of new blood vessels from preexisting ones [16] and may also contribute to metabolic processes. The VEGF concentration has been reported to correlate with BMI and is higher in overweight and obese patients [17, 18]. There is evidence of a positive correlation between VEGF-A, BMI, and waist circumference [19]. This is in contrast to the results of another study where obesity was associated with low VEGF, resulting in insufficient blood vessel development in the adipose tissue and local adipocyte hypoxia [20].

*Matrix metalloproteinases* (MMPs) are a group of endopeptidases whose activity increases under the influence of ongoing inflammation or certain growth factors and is inhibited by tissue inhibitors of metalloproteinases (TIMPs) [21]. MMPs play an important role in angiogenesis [22] and wound healing [23], and they take part in the modulation of adipogenesis [24]. MMP-1 seems to be involved in a process that facilitates the development of adipose tissue and, consequently, leads to obesity [25]. Moreover, plasma concentrations of MMP inhibitor (TIMP-1) are significantly higher in obese patients, and weight loss leads to a drop in TIMP-1 concentration [26]. The plasma levels of MMP-2 and MMP-9 are elevated in patients with metabolic syndrome, with or without diabetes mellitus [27].

The above-described chemical markers have only been analyzed in the adult population. In view of the increased risk of cardiometabolic complications in TS patients, we endeavored to bridge the gap by analyzing the concentrations of selected markers in normal-weight TS girls and comparing them with healthy girls.

## 2. Subjects and Methods

Twenty-eight patients were enrolled into this prospective study. The study group included 17 patients with Turner syndrome (TS), confirmed by karyotyping with routine G-banding according to the recommendations of the American College of Medical Genetics. All the patients in the study group were treated with recombinant growth hormone. The control group consisted of 11 girls with nonpathologic short stature and normal karyotype.

### 2.1. Clinical Phenotype of Study Participants

The detailed anthropometrical analysis was based on weight and height measurements, along with body mass index (BMI) calculation, using the standard formula of weight (kg) divided by height (m) squared. Weight was measured with a Seca scale with a precision of 100 g, and height with a Harpenden stadiometer with a graduation of 0.1 cm. A BMI above the 95^th^ percentile was classified as obesity, while a BMI between the 85^th^ and 95^th^ percentile as overweight. The height was expressed as standardized values (height standard deviation score (hSDS)) based on the growth chart for healthy Polish girls [28]. hSDS was calculated using the following formula: hSDS = child′s height − height for 50 pc/0.5∗(height 50 pc − height 3 pc). Given a participant's age, sex, BMI, and appropriate reference standard, the BMI *Z*-score was calculated using the Pediatric *Z*-Score Calculator (from the website of the Children's Hospital of Philadelphia, Research Institute: http://stokes.chop.edu/web/zscore/). In addition, the patients' bone age was determined based on the X-ray of the nondominating hand using the Greulich-Pyle Atlas [29]. The Tanner scale was used for puberty assessment [30].

### 2.2. Biochemical Phenotype of Study Participants

Morning fasting venous blood samples were collected to measure MMP-1 (matrix metalloproteinase-1), MMP-2 (matrix metalloproteinase-2), TIMP-1 (tissue inhibitor of metalloproteinase-1), MMP-9 (matrix metallopeptidase-9), BDNF (brain-derived neurotrophic factor), GDNF (glial cell line-derived neurotrophic factor), and VEGF (vascular endothelial growth factor). The concentrations of these markers were determined by sandwich ELISA, using kits distributed by the R&D systems. The concentrations of fT4 (free thyroxine), TSH (thyroid-stimulating hormone), ALT (alanine transaminase), AST (aspartate transaminase), and IGF-1 (insulin-like growth factor 1) were also determined. Serum concentrations of fT4 and TSH were measured with a chemiluminescent immunometric assay (IMMULITE 2000 Free T4 and IMMULITE 2000 Third Generation TSH, respectively; Siemens). Alanine and aspartate aminotransferase activity in the serum was assessed according to the International Federation in Clinical Chemistry (Beckman Coulter).

## 3. Statistical Analysis

Data processing and statistical analyses were performed using Statistica 13 PL software. *p* value < 0.05 was considered significant. The Shapiro-Wilk test was used to verify the normality of variables. Comparisons between two groups were performed using the Mann–Whitney *U* test. Regression analysis was used to investigate the correlation between quantitative variables. All results were reported as mean ± standard deviation (SD).

The study was conducted according to the Declaration of Helsinki and approved by the Ethics Committee of the Medical University of Silesia (resolution number KNW/0022/KB1/162/15/16). Written informed consent was obtained from each participant aged over 16, a parent, or a legal guardian.

## 4. Results

The baseline clinical characteristics of all the study participants are presented in Table 1. The groups did not differ in chronological or bone age. There were no differences in mean weight, although the *Z*-score BMI was higher in the study group. One control and two patients in the study group were overweight. None of the participants had a BMI > 95 pc (Table 2). The examined girls had comparable height and hSDS. According to the Tanner scale, 4 controls and 8 study patients started puberty (Tanner > B2), of whom 5 started spontaneously and 3 after the induction of estrogen replacement therapy. In all of the remaining participants, the onset of puberty has not been recorded. Regarding the study group, monosomy 45,X was confirmed in 8 patients, while in 9 patients, non-45,X karyotype was found.

The biochemical characteristics of both groups are presented in Table 3. The analyzed groups differed in mean baseline concentrations of MMP-1 and BDNF, which were significantly lower in the control group (both *p* < 0.05, with a probability of detecting a true effect of 0.8). Regression analysis, conducted in all 28 participants, revealed a significant correlation between MMP-1 concentrations and *Z*-score BMI, ALT activity, and BDNF and IGF-1 levels. The remaining correlations between MMP-1 and clinical and biochemical variables were not statistically significant (*p* > 0.05) (Table 4).

BDNF values correlated positively with *Z*-score BMI, TIMP-1, MMP-1, and MMP-9. A negative correlation was observed between BDNF and fT4, although there were no differences in fT4 between the control group and the study group (*p* > 0.05). There were no other significant correlations between BDNF and the remaining clinical and biochemical variables (*p* > 0.05) (Table 5).

Heart defects were observed in 11 patients in the study group: conduction disturbances, coarctation of the aorta, mitral regurgitation, bicuspid aortic valve, and patent foramen ovale (Table 6). Six patients in the study group were free from any heart abnormalities. Three of the four highest BDNF values were observed in patients with conduction disturbances.

Two patients had hypertension; thus, no conclusions could be drawn.

All patients in the study group received rGH with a median dose of 47–66 *μ*g/kg/day. None of the markers correlated with the dose of rGH; however, there was a positive correlation between MMP-1 and IGF-1 (*r* = 0.46, *p* = 0.01), as well as a correlation between IGF-1 and the dose of rGH (*r* = 0.92, *p* < 0.01).

## 5. Discussion

To the best of our knowledge, this is the first study to discuss selected neurotrophins, VEGF, and matrix metalloproteinases as potential prognostic makers of cardiometabolic complications in girls with Turner syndrome.

Besides many other functions, the examined markers seem to be related to metabolic parameters. Numerous studies have shown that the concentrations of selected markers are related to metabolic disorders and obesity, more frequently observed in patients with TS, even in childhood. Accordingly, we searched for the differences in the concentrations of individual markers between the study group and the control group, even in the presence of normal body weight. The control group was composed of healthy girls with short stature, whose chronological and bone age was comparable to that of the study group.

Our analysis revealed no significant differences between the study group and the control group in MMP-2, TIMP-1, MMP-9, GDNF, and VEGF concentration ranges. Since the concentrations of MMP-2 and MMP-9 are higher in patients with the metabolic syndrome [27] and TIMP-1 is significantly higher in obese patients [26], we assumed that the higher concentrations of the analyzed markers are the consequence rather than the cause of cardiometabolic complications.

We can expect that the absence of differences in GDNF concentrations between the study group and the control group results from the suspicion that plasma GDNF concentrations do not reflect the concentrations of GDNF in tissues involved in regulating energy metabolism. Although the expression of GDNF is increased in glial cells, liver, and white and brown adipose tissue in transgenic mice overexpressing GDNF, the plasma concentration does not change [31].

We observed no differences in VEGF concentrations between the study group and the control group. So far, studies on the association of VEGF with obesity or metabolic complications have given contradictory results [17, 18, 20]. Studies on VEGF concentrations in TS are very limited and focus mainly on the role of VEGF overexpression during fetal life, leading to fetal hydrops, congenital heart defects, or short stature and gonadal dysgenesis [32]. Further studies are needed to clarify the relationship between this growth factor and the risk of metabolic disturbances.

Our study showed differences in the mean values of BDNF between the study group and the control group, with higher concentrations in the first. There is no data on BDNF concentrations in girls with TS available in literature. However, the results of a study assessing BDNF in an adult TS population proved that BDNF concentrations were significantly higher (two-fold) in those patients compared to healthy women [33]. It was a surprising finding, since the majority of TS patients in that experiment were overweight or obese, and the mean value of BMI in the study group was significantly higher than that in the control group. Those results are in contrast with the results of other studies, which report a reduction of neurotrophins in the presence of metabolic syndrome components. BDNF correlated positively with testosterone levels in women with TS; accordingly, it was hypothesized that androgens in women with nonfunctional ovaries may be one of the main regulators of plasma BDNF levels. Some reports claim that BDNF concentrations lower with age [8]; hence, decreased BDNF could be considered a marker of aging. It could therefore be presumed that the lack of estrogen observed in girls with TS may resemble the menopausal status and result in lowered BDNF levels in patients with TS. A question arises as to the influence of estrogen-androgen imbalance on the higher BDNF concentration observed in our patients; however, this was not the purpose of our study and shall be discussed elsewhere.

The regression analysis conducted for all our participants revealed a positive correlation between BDNF and *Z*-score BMI. This stands in contrast to previous findings, according to which BDNF concentration correlates negatively with body weight [7], and to the results of the aforementioned study by Czyzyk et al., in which no statistical significance was found between BDNF concentration and BMI [33]. It might stem from the fact that our research was conducted on a small group of patients and requires confirmation with the participation of a larger number of patients. What is more, in the study conducted by Lommatzsch et al. [7], it was found that platelet BDNF levels are lower in women than in men and change during the menstrual cycle. Therefore, the dependencies we have identified require further investigation in connection with the aforementioned influence of estrogen-androgen imbalance in TS patients.

In our study, we also found differences in plasma MMP-1 concentrations between the study group and the control group. As with BDNF concentrations, also concentrations of metalloproteinase were higher in the study group. The regression analysis demonstrated a positive correlation between MMP-1 and *Z*-score BMI, possibly confirming a link between MMP-1 and body weight. MMP-1 is involved in tissue remodeling during adipose tissue expansion in obesity [25]; however, it is uncertain whether plasma MMP-1 concentration varies depending on BMI [26].

Elevated levels of MMP-1 have been observed in human carotid atherosclerosis [25], possibly suggesting a higher risk of carotid artery atherosclerosis in girls with TS. What is more, increased local and systemic MMP-1 activation caused by CRP stimulation seems to be associated with inflammation and plaque vulnerability. Taking into consideration the elevated cardiovascular risk in TS [34], examining the relationship between the concentration of this metalloproteinase and the severity of atherosclerotic lesions in TS appears to be of importance.

The results presented here are unique in the context of the study group, patients' age, and the aim of comparing selected metabolic markers in nonobese patients. We are aware that one of the most important limitations of our study is the small number of patients, making it more suitable as a pilot study. We are carrying out further studies on a larger group of patients to assess all the components of the metabolic syndrome and the concentrations of the listed markers.

## Figures and Tables

**Table 1:** Clinical characteristics of the control group and the study group.

	Control group (*n* = 11)	Study group (*n* = 17)	*p* value
Age (years)	9.2 ± 3.2	11.0 ± 3.6	0.14
Bone age (years)	8.4 ± 2.7	10.2 ± 2.9	0.13
Height (cm)	127.9 ± 14.4	129.6 ± 20.2	0.64
hSDS	−2.7 ± 0.7	−2.6 ± 1.0	0.69
Weight (kg)	25.6 ± 7.4	32.7 ± 12.8	0.10
BMI *Z*-score	−1.1 ± 1.3	−0.1 ± 0.8	0.03

Mann–Whitney *U* test. Data are presented as mean ± SD. TS: Turner syndrome; hSDS: height standard deviation score; BMI *Z*-score: body mass index *Z*-score.

**Table 2:** BMI distribution in groups.

	Control group (*n* = 11)	Study group (*n* = 17)
Underweight	4	1
Normal	6	14
Overweight	1	2
Obese	0	0

**Table 3:** Biochemical characteristics of the control group and the study group.

	Control group (*n* = 11)	Study group (*n* = 17)	*p* value
MMP-1 (ng/ml)	3.8 ± 2.3	11.4 ± 6.8	<0.001
MMP-2 (ng/ml)	239.5 ± 31.6	258.9 ± 64.0	0.47
TIMP-1 (ng/ml)	216.2 ± 67.8	242.9 ± 74.7	0.27
MMP-9 (ng/ml)	351.0 ± 141.5	421.2 ± 186.7	0.29
BDNF (ng/ml)	24.1 ± 6.3	38.4 ± 13.2	0.001
GDNF (ng/ml)	5.7 ± 13.9	3.1 ± 2.5	0.45
VEGF (ng/ml)	125.5 ± 120.3	69.5 ± 66.8	0.18
fT4 (ng/dl)	1.3 ± 0.1	1.3 ± 0.2	0.38
TSH (mIU/l)	2.4 ± 0.9	3.1 ± 1.6	0.37
ALT (IU/l)	14.7 ± 4.1	17.7 ± 3.1	0.06
AST (IU/l)	34.3 ± 10.0	31.2 ± 10.0	0.39
IGF-1 (ng/ml)	172.1 ± 110.3	549.2 ± 264.5	<0.001

Mann–Whitney *U* test. Data are presented as mean ± SD. hSDS: height standard deviation score; MMP-1: matrix metalloproteinase-1; MMP-2: matrix metalloproteinase-2; TIMP-1: tissue inhibitor of metalloproteinase-1; MMP-9: matrix metallopeptidase 9; BDNF: brain-derived neurotrophic factor; VEGF: vascular endothelial growth factor; fT4: unbound T_4_; TSH: thyroid-stimulating hormone; ALT: alanine transaminase; AST: aspartate transaminase; IGF-1: insulin-like growth factor 1.

**Table 4:** MMP-1 regression analysis of all study participants.

	BMI *Z*-score	ALT (IU/l)	BDNF (ng/ml)	IGF (ng/ml)
MMP-1 (ng/ml)	*r* = 0.36 *p* = 0.047	*r* = 0.44 *p* = 0.025	*r* = 0.45 *p* = 0.007	*r* = 0.46 *p* = 0.011

MMP-1: matrix metalloproteinase-1; BMI *Z*-score: body mass index *Z*-score; ALT: alanine transaminase; BDNF: brain-derived neurotrophic factor; IGF-1: insulin-like growth factor 1.

**Table 5:** BDNF regression analysis of all study participants.

	BMI *Z*-score	TIMP-1 (ng/ml)	MMP-1 (ng/ml)	MMP-9 (ng/ml)	fT4 (ng/l)
BDNF (ng/ml)	*r* = 0.48 *p* = 0.013	*r* = 0.52 *p* = 0.001	*r* = 0.45 *p* = 0.007	*r* = 0.46 *p* = 0.005	*r* = −0.43 *p* = 0.03

BDNF: brain-derived neurotrophic factor; BMI *Z*-score: body mass index *Z*-score; TIMP-1: tissue inhibitor of metalloproteinase-1; MMP-1: matrix metalloproteinase-1; MMP-9: matrix metallopeptidase 9; fT4: unbound T_4_.

**Table 6:** Study group characteristics.

Study group (*N* = 17)	*N* (%)
Type of cardiopathy	
None/CD/CoA	6 (35.3)/6 (35.3)/2 (11.8)
MR/BAV/PFO	1 (5.9)/1 (5.9)/1 (5.9)
Hypertension	
Yes	2 (11.8)
No	15 (88.2)
Hypothyroidism	
Yes	1 (5.9)
No	16 (94.1)
History of hypothyroidism	
Yes	6 (35.3)
No	11 (64.7)

CD: conduction disturbances; CoA: coarctation of the aorta; MR: mitral regurgitation; BAV: bicuspid aortic valve; PFO: patent foramen ovale.

## Data Availability

The results are stored in the computer memory in a form that prevents the identification of people qualified for the test. Data are available on request. To obtain access to data, please contact the author for correspondence.
